# Endothelin signaling regulates mineralization and posttranscriptionally regulates SOST in TMOb cells via miR 126‐3p

**DOI:** 10.14814/phy2.13088

**Published:** 2017-02-24

**Authors:** Michael G. Johnson, Kathryn Konicke, Jasmin Kristianto, Anne Gustavson, Rachel Garbo, Xiaohu Wang, Baozhi Yuan, Robert D. Blank

**Affiliations:** ^1^ Geriatrics ResearchEducation and Clinical CenterWilliam S. Middleton Veterans Affairs HospitalMadisonWisconsin; ^2^ Division of EndocrinologyDepartment of MedicineUniversity of Wisconsin‐MadisonMadisonWisconsin; ^3^ Medical ServiceClement J. Zablocki Veterans Affairs Medical CenterMilwaukeeWisconsin; ^4^ Division of EndocrinologyDepartment of MedicineMedical College of WisconsinMilwaukeeWisconsin

**Keywords:** Endothelin‐1, endothelin‐converting enzyme‐1, mineralization, miR 126‐3p, osteoblast

## Abstract

Previously, our laboratory identified *ECE‐1*, encoding endothelin‐converting enzyme‐1 (ECE‐1), as a positional candidate for a pleiotropic quantitative trait locus affecting femoral size, shape, and biomechanical performance. We hypothesized that endothelin‐1 (ET‐1) signaling promotes osteogenesis. Exposure of immortalized mouse osteoblast (TMOb) cells to big ET‐1 increased mineralization. Following big ET‐1 treatment, we measured the secretion of insulin‐like‐growth factor‐1 (IGF1), dickkopf‐homolog‐1 protein 1 (DKK1), and sclerostin (SOST). In each case, big ET‐1 signaling changed secretion in a manner that favored increased osteogenic activity. Treatment with ECE‐1, endothelin receptor A (EDNRA), or WNT receptor antagonists inhibited the big ET‐1‐mediated increase in mineralization. In the presence of big ET‐1, message levels of *Runx2*,* Igf1*,* Dkk1,* and *Sost* are uncoupled from protein production, suggesting posttranscriptional regulation. To evaluate the role of big ET‐1 in normal bone physiology, we inhibited EDNRA signaling during mineralization in the absence of exogenous ET‐1. EDNRA blockade reduced mineralization, decreased IGF1, and increased DKK1 and SOST secretion, responses opposite to those induced by exogenous big ET‐1. Pharmacological and siRNA knockdown to inhibit ECE‐1 reduced mineralization and IGF1 secretion with decreasing DKK1 and decreasing or stable SOST secretion, suggesting a further, unknown role of ECE‐1 in osteoblast maturation. Previously we identified miR 126‐3p as a potential ET‐1‐responsive regulator of SOST in murine cells. Overexpression of miR126‐3p increased mineralization in TMOb cells and decreased SOST secretion. Osteoblasts express the ET‐1 signaling pathway and ET‐1 signaling is necessary for normal osteoblast differentiation and mineralization, acting through regulation of miRs that target osteogenic molecules.

## Introduction

Previous gene targeting studies showed that endothelin‐1 (ET‐1) signaling is critical in development. Homozygous *Edn1* ^*−/−*^ and *Ednra* ^*−/−*^ mice have cardiovascular outflow tract abnormalities and craniofacial malformations, resulting in embryonic or perinatal death (Kurihara et al. 1994; Clouthier et al. 1998; Yanagisawa et al. 1998; Kedzierski and Yanagisawa 2001). Homozygous *ECE‐1* ^*−/−*^ mice have the combined phenotypes of homozygous *Edn1* ^*−/−*^, *Ednra* ^*−/−*^, *Edn3* ^*−/−*^, and *Ednrb* ^*−/−*^ mice, but in more severe form (Yanagisawa et al. 1998). These studies showed the importance of endothelin‐converting enzyme‐1 (ECE‐1)*‐*dependent ET‐1 signaling in development. Although other endogenous metalloproteases can cleave big ET‐1 to active ET‐1, they are unable to compensate for the absence of ECE‐1 function (Kurihara et al. 1994; Clouthier et al. 1998; Yanagisawa et al. 1998). Taken together, these data demonstrate that the time and place of ET‐1 activation by ECE‐1 are crucial for normal development (Kurihara et al. 1994; Clouthier et al. 1998; Yanagisawa et al. 1998).

ECE‐1 is a membrane‐bound extracellular metalloprotease that catalyzes the conversion of the biologically inactive big ETs to their active forms. There are three ETs, each of which has distinct roles in development and physiology (Chakravarti 1996; Edery et al. 1996; Bruzzi et al. 1997; Bagnato and Natali 2004; Bagnato and Rosano 2008; Barton and Yanagisawa 2008; Davie et al. 2009; Bagnato et al. 2011). ET‐1 is a short‐lived, potent, autocrine/paracrine signaling molecule that was first recognized for its vasoconstrictive effects (Yanagisawa et al. 1988). The effects of ET‐1 in the context tumor pathology of bone are well known. ET‐1 signaling promotes concordant synthesis of woven bone and tumor growth, a constellation of findings often referred to as osteoblastic metastasis (Berruti et al. 1999; Guise et al. 2003, 2005, 2006; Mohammad and Guise 2003; Yin et al. 2003; Clines et al. 2007; Tucci et al. 2009). Multiple reports confirm that tumor‐produced ET‐1 enhances osteoblast mineralization in this setting and that exogenous mature ET‐1 can do so in vitro, but the role of ET‐1 signaling in normal bone remains largely unexplored (Orzechowski et al. 1997; von Schroeder et al. 2003; Clines et al. 2007, 2011).

Previous work in our laboratory using the recombinant congenic mice HcB‐8 and HcB‐23 identified *ECE‐1*, the gene encoding ECE‐1, as a positional candidate for a pleiotropic quantitative trait locus influencing bone strength and size in skeletally mature mice (Saless et al. 2009, 2010, 2011a,b; Kristianto et al. 2016). In these mice, higher expression of ECE‐1 is associated with the presence of larger, stronger, and denser bones (Wang et al. 2013). We hypothesized that big ET‐1 signaling promotes osteoblast differentiation and mineralization in normal physiology. Further, we hypothesized that inhibition of endothelin receptor A (EDNRA) and/or ECE‐1 in vitro would block the effects of big ET‐1 supplementation and that inhibition of ET‐1 signaling in the absence of supplemental big ET‐1 would decrease mineralization. To test these ideas, we exposed immortalized mouse pre‐osteoblasts (TMOb) cells (Xiao et al. 1998) to exogenous big ET‐1 and pharmacologically blocked the activity of EDNRA and ECE‐1 and used siRNA to knock down *Ece‐1* expression ± big ET‐1. The experiments showed that ECE‐1‐dependent ET‐1 signaling directly influenced normal osteoblast differentiation and mineralization by decreasing the secretion of the WNT signaling inhibitors sclerostin (SOST) and dickkopf‐homolog‐1 (DKK1) and increasing secretion of insulin‐like‐growth factor‐1 (IGF1).

Previously, we demonstrated that SOST secretion and message levels are uncoupled and identified miR 126‐3p as a potential regulator of translation of the WNT inhibitor SOST (Johnson et al. 2014). Big ET‐1 treatment of TMOb cells increased the levels of mir 126‐3p during mineralization 121x. MiR 126‐3p was first identified as an important factor in angiogenesis (Fish et al. 2008; Kuhnert et al. 2008; Wang et al. 2008; Cao et al. 2015). MiR 126‐3p represses two negative regulators of VEGF signaling, sprouty‐related protein‐1, and phosphatidylinositol‐3‐kinase regulatory subunit 2, which leads to increased VEGF signaling and increased angiogenesis (Fish et al. 2008; Kuhnert et al. 2008; Wang et al. 2008; Cao et al. 2015). To determine the mechanism, we constructed stable TMOb cell lines overexpressing miR 126‐3p, its inhibitor, and scrambled controls. We demonstrated that miR 126‐3p posttranscriptionally regulates SOST secretion and TMOb mineralization. Our study identifies regulation of miR 126‐3p as a mechanism by which cross talk between the ET‐1 and WNT signaling pathways affects mineralization of TMOb osteoblasts.

## Experimental Procedures

### Cell culture

TMOb cells were cultured in *α*‐MEM and 10% fetal bovine serum with 1% Pen/Strep (Xiao et al. 1998). Mineralization medium also included 50 *μ*g/mL of vitamin C and 10 mmol/L *β*‐glycerol phosphate as previously described (Xiao et al. 1998). When present, the concentration of big ET‐1 (Sigma‐Aldrich, St. Louis, MO) was 25 ng/mL. The following concentrations of pharmacologic agents were applied: 10 *μ*mol/L BQ‐123 (A.G. Scientific, San Diego, CA), 20 *μ*mol/L phosphoramidon, a nonspecific ECE‐1 inhibitor (Sigma‐Aldrich), and 30 ng/mL SOST (R&D Systems, Minneapolis, MN) (Ishikawa et al. 1992; Matsumaru et al. 1998). Cells were grown to confluence in 25‐cm^2^ flasks and harvested using Tryple Select (Invitrogen, Carlsbad, CA). Cells harvested from four flasks were pooled in 15 mL of growth medium. 0.1 mL of cell suspension/well was added to six‐well plates containing 2 mL of nonmineralization medium ± big ET‐1. The cells were grown for 6 days with one change in nonmineralization medium ± big ET‐1 after 3 days. After 6 days, the medium was changed to mineralization medium ± big ET‐1. Day 0 was defined as the day on which mineralization medium was first added to the cells. Experiments were continued for 15 days thereafter, with refeeding every 3 days. At days 0, 3, 6, 9, 12, and 15, conditioned medium, RNA, and protein were collected from each well of a six‐well plate and stored at −80°C until analyzed.

### Alizarin red staining

On day 15, we measured mineralization of TMOb cells by alizarin red staining (Xiao et al. 1998). Briefly, cells were fixed with 1 mL 70% ethanol for 1 h, stained with 1 mL 20 mmol/L alizarin red pH 4.0 for 20 min (Sigma‐Aldrich), and washed 5X with double‐distilled water and once with PBS. After drying, wells were photographed at 10× magnification on a dissecting microscope using a MotiCam 1 camera (ThermoFischer Scientific, Rockford, IL). SigmaScan (Systat Software International, San Jose, CA) was used to count the number of red pixels per field. Five random fields per well were quantitated and averaged. Five fields from each well of a six‐well plate were analyzed and used to calculate the average number of pixels per field for each time point. Representative images of a well from each treatment (i.e., from an individual six‐well plate and a separate photograph) are shown in figures. Results were analyzed by one‐way analysis of variance (ANOVA).

### Proliferation assay

Proliferation was measured using the CyQuant proliferation assay (Invitrogen) according to the manufacturer's protocol. Cells were grown to confluence in 25‐cm^2^ flasks, harvested with Tryple Select, and suspended in 10 mL of nonmineralization medium. The suspended cells were diluted 1:10 in each treatment medium, and 0.2 mL was plated in quadruplicate on sterile 96‐well plates. One plate was prepared for each time point. Plates were grown for 6, 24, 48, and 72 h. Results were analyzed by two‐way repeated‐measures ANOVA.

### Real‐time PCR

TMOb cells grown on six‐well plates (±big ET‐1) were bathed in 1 mL TRIzol (Invitrogen) on days 0, 3, 6, 9, 12, and 15. The average of each well of a six‐well plate was determined for each time point. Technical replicates for each sample were performed in triplicate and repeated at least twice. Plates were shaken for 10 min at room temperature and RNA was isolated from the TRIzol reagent using the RNeasy mini kit (Qiagen, Valencia, CA) according to the manufacturer's protocol. RNA concentrations were determined using a Synergy 2 plate reader (BioTek, Winooski, VT) according to the manufacturer's Take 3 protocol. Forty microliters of cDNA per sample was synthesized using the iScript cDNA synthesis kit (Bio‐Rad, Hercules, CA) and 2 *μ*g of total RNA according to the manufacturer's protocol. Messenger RNA levels were measured by the ^ΔΔ^ *C*_*t*_ method using GAPDH as a reference. Real‐time PCRs were performed in a StepOne RT‐PCR instrument (Life Technologies, Carlsbad, CA) using TaqMan (Life Technologies) assays according to the manufacturers' instructions. Assay IDs are shown in Table 1. Time courses were analyzed by two‐way repeated‐measures ANOVA. The identification of which ET signaling axis genes were present in TMOb cells was performed by dichotomous absence/presence qPCR.

**Table 1:** Summary of genes analyzed by qPCR and their assay identification numbers

Gene	Assay ID
*Edn1*	Mm00438656_m1
*Edn2*	Mm00432983_m1
*Edn3*	Mm00432986_m1
*Ece‐1*	Mm01187104_m1
*Ednra*	Mm01243710_m1
*Ednrb*	Mm00432989_m1
*Runx2*	Mm00501584_m1
*Dkk1*	Mm00438422_m1
*Sost*	Mm00470479_m1
*Igf1*	Mm00439560_m1
*Gapdh*	Mm99999915_g1

### *Ece‐1* siRNA transfections

A mineralization assay of TMOb cells requires 15 days of culture after the media is changed from proliferation to mineralization. Therefore, transfections of *ECE‐1* siRNA were performed on days 0 and 6 to knock down *ECE‐1* message levels over the course of the experiment. Transfections were performed using Lipofectamine (Invitrogen) according to the manufacturer's protocol. *ECE‐1* siRNA (Invitrogen) or Stealth RNAi negative control (Invitrogen) were transfected into TMOb cells at a final concentration of 5 nmol/L. Efficiency of siRNA transcript knockdown was assessed 3 days after the day 0 transfection using the KDalert GAPDH Assay Kit according to the manufacturer's protocol (Life Technologies) and by Western analysis of ECE‐1 protein levels on day 15, 9 days after day 6 transfection and analyzed by *t*‐test. At days 0, 3, 6, 9, 12, and 15, conditioned medium was collected from each well of a six‐well plate and stored at −80°C until analyzed. Results from all six wells were averaged to estimate the value for a time point. Day 15 plates were stained with alizarin red and analyzed as described and analyzed by one‐way ANOVA.

### Western blots

Protein concentrations were determined via Bradford Assay (Bio‐Rad). Proteins were separated by sodium dodecyl sulfate–polyacrylamide gel electrophoresis (SDS‐PAGE) (10% gel) and transferred to nitrocellulose membranes. Each time point for a treatment was done in sextuplicate, and each time point ± big ET‐1 was run on a separate gel. The blots were blocked and then incubated with ECE‐1, RUNX2, or *β*‐actin antibody (Santa Cruz Biotechnology, Santa Cruz, CA) at a 1:200 dilution. Blots were washed and next incubated with a 1:2000 dilution of Rabbit Anti‐Goat HRP Conjugate (Santa Cruz Biotechnology). The immunoreactive bands were visualized via the ECL detection system (Amersham, Buckinghamshire, U.K.) and analyzed densitometrically with Adobe Photoshop CS4 Extended (Adobe Systems Inc., San Jose, CA). Target protein intensities were normalized to *β*‐actin. For each treatment, samples from day 3, 6, 9, 12, and 15 (*n* = 6 for each treatment, 12 total samples) were analyzed for RUNX2 expression (±big ET‐1) on separate gels for each time point. Controls were normalized to 1 during analysis. RUNX2 production by control and ET‐1‐treated TMOb cells were compared by *t*‐test for each time point. Each figure shows one representative lane of RUNX2 or ECE‐1 and it is the corresponding exposure of *β*‐actin.

### ELISA

We analyzed protein levels of DKK1, SOST, and IGF1 from days 0, 3, 6, 9, 12, and 15 in conditioned cell culture media using Quantikine HS or DuoSet ELISA kits (R&D Systems) according to the manufacturer's protocol. For each treatment, a time point consisted of a six‐well plate. Media from each well was analyzed in duplicate, and the results were averaged. Results from all six wells of a time point were averaged to estimate the value for a time point. Differences in protein secretion were analyzed by two‐way repeated‐measures ANOVA.

### Hydroxyproline assay

Total hydoxyproline production of TMOb cells ± big ET‐1 was assessed on days 0, 3, 6, 9, 12, and 15 using the Hydroxyproline Assay Kit (Sigma‐Aldrich) on material scraped from each well of a six‐well plate according to the manufacturer's instructions and normalized to protein concentration. Results from all six wells of a time point were averaged to estimate the value for a time point and were analyzed by two‐way repeated‐measures ANOVA.

### Lentivirus constructs

We obtained lentivirus constructs of miR 126‐3p, its mimic, and their scrambled controls (GeneCopoeia, Rockville, MD) and created stable TMOb cell lines according to the manufacturer's instructions. To test the effect of miR 126‐3p and its inhibitor on mineralization and SOST secretion in TMOb cells, we cultured all four stable cell lines and a normal control ± big ET‐1. At days 0, 3, 6, 9, 12, and 15, conditioned medium was collected from each well of a six‐well plate and stored at −80°C until analyzed. Media from all six wells of a time point were tested in duplicate and averaged. Results from all six wells were averaged to estimate the value for a time point. A second day 15 plate was stained with alizarin red and analyzed as described. Mineralization was analyzed by one‐way ANOVA.

### Statistical analysis

We used SigmaStat 3.5 (Systat Software International) to perform statistical analysis. Mineralization results were analyzed by *t*‐test for comparisons of two groups or ANOVA for comparisons of multiple groups. Measures of protein production or secretion over time were analyzed by two‐way repeated‐measures ANOVA and used the Holm–Sidak method to determine differences among test groups. For each time point for each treatment, we used an *n* of 6. If the data could not be transformed to satisfy the assumptions of normality and equal variance, we used ANOVA on ranks to compare groups. All values were reported as mean ± SEM.

## Results

### Mineralization of TMOb cells ± big ET‐1 in the presence of SOST, ECE‐1, and EDNRA inhibitors

**Figure 1:**
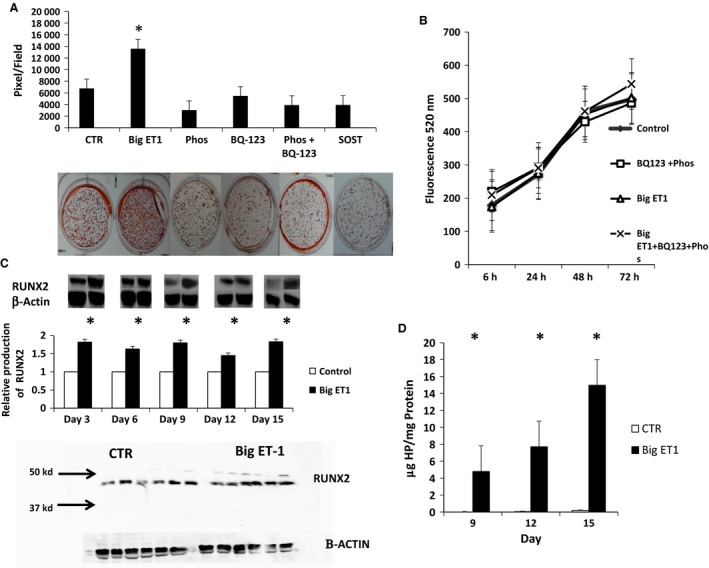
Mineralization and proliferation of TMOb cells ± big ET‐1. (A) Mineralization of TMOb cells in the presence of BQ‐123 (endothelin receptor A [ENDRA] inhibitor), phosphoramidon (ECE‐1 inhibitor) and SOST (LRP5/6 inhibitor). Each picture is a representative well cut from a picture of a six‐well plate that makes up a time point. Mineralization in the big ET‐1 group was significantly greater (*P* < 0.05) than all other groups. Big ET‐1 was present in all inhibitor groups. There were no significant differences between inhibitor groups. Degree of mineralization is the average of six samples, and the data were analyzed by one‐way ANOVA. Each image is a representative well cut from a picture of a six‐well plate that makes up a treatment. (B) Proliferation of TMOb cells ± big ET‐1 and ±phosphoramidon/BQ‐123. There were no significant differences in cell growth between the no treatment control and any of the treatment groups. Each time point is the average of four samples and was analyzed by two‐way repeated‐measures ANOVA. (C) The presence of big ET‐1 increased the production of RUNX2 by Western blot analysis in TMOb cells during mineralization by ~80% on days 3, 6, 9, 12, and 15 (*P* < 0.05 for each day). Each time point is the average of six samples, and each group was analyzed by *t*‐test. The figure shows representative lanes from each set of six samples, using the same lane for the RUNX2 and *β*‐actin. Each time point was run on a separate gel and controls were normalized to 1 for each time point before statistical analysis. (D) The presence of big ET‐1 increased the production of collagen as measured by hydroxyproline content in matrix produced by TMOb cells during mineralization on days 9, 12, and 15 (*P* < 0.001 for each day). Each time point is the average of six samples. Data were analyzed by two‐way repeated‐measures ANOVA. TMOb, immortalized mouse pre‐osteoblasts; ET‐1, endothelin‐1; ECE‐1, endothelin‐converting enzyme‐1; SOST, sclerostin. * denotes significance *P* < 0.05.

Previously, we demonstrated that TMOb cells exposed to exogenous big ET‐1 showed increased mineralization (Orzechowski et al. 1997). This greater mineralization suggested that exposure to big ET‐1 caused increased synthetic activity of individual osteoblasts, increased osteoblast proliferation, increased osteoblast differentiation, or a combination of these. To evaluate the mechanism of big ET‐1's effect on mineralization, we added phosphoramidon (ECE‐1 inhibitor), BQ‐123 (EDNRA inhibitor), or SOST (bone‐specific LRP5/6 inhibitor) to mineralization media containing big ET‐1. Each picture is a representative well cut from a picture of a six‐well plate that makes up a time point. Figure 1A demonstrates that the presence of each inhibitor blocked the effect of big ET‐1 (*P* < 0.05) as analyzed by one‐way ANOVA. These findings demonstrated that the change in mineralization due to big ET‐1 exposure was dependent on ECE‐1, EDNRA, and WNT signaling.

### Proliferation of TMOb cells ± big ET‐1

To determine whether increased proliferation contributed to the increase in mineralization arising from big ET‐1 exposure, we measured TMOb cells' proliferation in the presence or absence of ET‐1 and the pharmacologic inhibitors phosphoramidon (ECE‐1) and BQ‐123 (EDNRA) and analyzed by two‐way repeated‐measures ANOVA. Figure 1B shows that big ET‐1 and the inhibitors did not affect proliferation, suggesting that accelerated differentiation or increased extracellular matrix deposition caused by increased metabolic activity, rather than increased cell number, accounted for the observed increase in mineralization.

### RUNX2 and collagen production in TMOb cells ± big ET‐1

To evaluate the effect of exogenous big ET‐1 on the mineralizing metabolism of TMOb cells, we measured the production of RUNX2, a master osteogenic transcription factor whose levels vary from high early during osteoblast differentiation to low in mature osteoblasts (Komori 2006), and hydroxyproline, an abundant constituent of type 1 collagen, which is the primary protein (~90% of the total protein) in bone extracellular matrix. Western blot analysis (Fig. 1C) shows a relative increase in RUNX2 production in mineralizing TMOb cells treated with big ET‐1 on days 3, 6, 9, 12, and 15 (*P* < 0.05 for each day). For each day, production of RUNX2 by control cells was normalized to 1 allowing for relative comparison of RUNX2 levels at each time point. Twelve samples, six each ± big ET‐1 were run on a single gel and analyzed by densitometry. Each time point was run on a separate gel and analyzed by *t*‐test. There were no comparisons of RUNX2 levels between time points. Figure 1C shows representative lanes of RUNX2 ± big ET‐1 and the corresponding exposure of *β*‐actin from each time point. A sample RUNX2 blot and equivalent *β*‐actin exposures are shown. Figure 1D shows that the presence of exogenous big ET‐1 increased the production of collagen as measured by hydroxyproline levels in mineralizing TMOb cells on days 9, 12, and 15 (*P* < 0.001 for each day). These data suggest that big ET‐1 causes an increase in differentiation and metabolic activity in mineralizing TMOb cells.

### Determination of the presence of ET signaling axis genes in TMOb cells during proliferation and mineralization

To determine the presence/absence of ET signaling axis genes, we used qPCR presence or absence experiments to detect ET signaling axis transcripts in TMOb cells on days 0, 3, 6, 9, 12, and 15 during mineralization. We were able to detect *ECE‐1*,* EdnRA*, and *Edn1* transcripts. We did not detect *EdnRB*,* Edn2*, or *Edn3*.

### Message levels of *Igf1*,* Dkk1,* and *Sost,* and secretion of IGF1, DKK1, and SOST in TMOb cells ± big ET‐1

**Figure 2:**
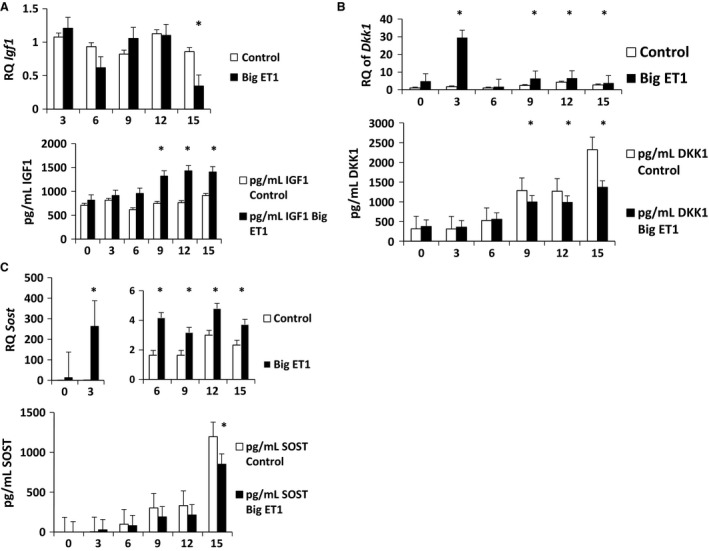
IGF1, DKK1, SOST secretion, and their mRNA levels in TMOb cells ± big ET‐1. Analysis by qPCR and ELISA. (A) Analysis of *Igf1* mRNA and secreted IGF1 in TMOb cells treated with exogenous big ET‐1. During mineralization, there was no difference in mRNA expression between treatments over time. There was a significant decrease in *Igf1* mRNA level in big ET‐1‐treated cells on day 15 (*P* < 0.05). There were significant differences between the control and big ET‐1 groups in IGF1 secretion in over time (*P* < 0.001) and significant increases in IGF1 protein secretion in the big ET‐1 group on days 6, 9, and 15 (*P* < 0.05) as analyzed by two‐way repeated‐measures ANOVA. (B) Analysis of *Dkk1* mRNA and secreted DKK1 in TMOb cells treated with exogenous big ET‐1. There were significant increases in *Dkk1* mRNA expression over time (*P* < 0.05) in the big ET‐1 arm on days 3, 9, 12, and 15 (*P* < 0.005 for each day). There were significant differences between the control group and the big ET‐1 group in treatment over time in the amount of secreted DKK1 protein (*P* < 0.001). There were significant decreases in the amount of DKK1 protein between the control group and the big ET‐1 group on days 9 (*P* < 0.05), 12 (*P* < 0.05), and 15 (*P* < 0.05) as analyzed by two‐way repeated‐measures ANOVA. (C) Analysis of *Sost* mRNA and secreted SOST protein in TMOb cells treated with exogenous big ET‐1. There were significant differences in *Sost* mRNA level between groups over time (*P* < 0.001). Big ET‐1 caused an increase in *Sost* transcription on days 3, 6, 9, 12, and 15 (*P* < 0.05 for each day). There was a significant decrease between the control and treatment groups in SOST secretion over time and a difference in secretion on day 15 (*P* < 0.001) as analyzed by two‐way repeated‐measures ANOVA. IGF1, insulin‐like‐growth factor‐1; DKK1, dickkopf‐homolog‐1; SOST, sclerostin; TMOb, immortalized mouse pre‐osteoblasts; ET‐1, endothelin‐1; ANOVA, analysis of variance. * denotes statistically significant difference.

To determine whether ET‐1 signaling transcriptionally regulates IGF1, DKK1, and SOST, we used real‐time PCR to measure message levels of *Igf1*,* Dkk1*, and *Sost*. We measured protein secretion of IGF1, DKK1, and SOST into the medium over the course of mineralization by ELISA. There was no difference in *Igf1* mRNA level between treatments (Fig. 2A). However, treatment with big ET‐1 increased IGF1 secretion during mineralization (Fig. 2A, *P* < 0.001), with significant increases in IGF1 secretion on days 6, 9, and 15 (*P* < 0.05). Similarly, treatment with big ET‐1 significantly decreased endogenous DKK1 and SOST secretion during mineralization (Fig. 2B and C, *P* < 0.001). There were significant increases in *Dkk1* mRNA levels over time (*P* < 0.05) in the big ET‐1‐treated cells on days 3, 9, 12, and 15 (*P* < 0.005 for each day). There were significant differences in *Sost* mRNA levels between groups over time (*P* < 0.001). Big ET‐1 caused an increase in *Sost* transcription on days 3, 6, 9, 12, and 15 (*P* < 0.05) for each day (Fig. 2B and C). These data show that I*gf1*,* Dkk1*, and *Sost* transcription and translation are uncoupled in TMOb cells in the presence of big ET‐1.

### Mineralization of TMOb cells and secretion of IGF1, SOST, and DKK1 in the presence of ECE‐1 and EDNRA inhibitors without the addition of exogenous big ET‐1

**Figure 3:**
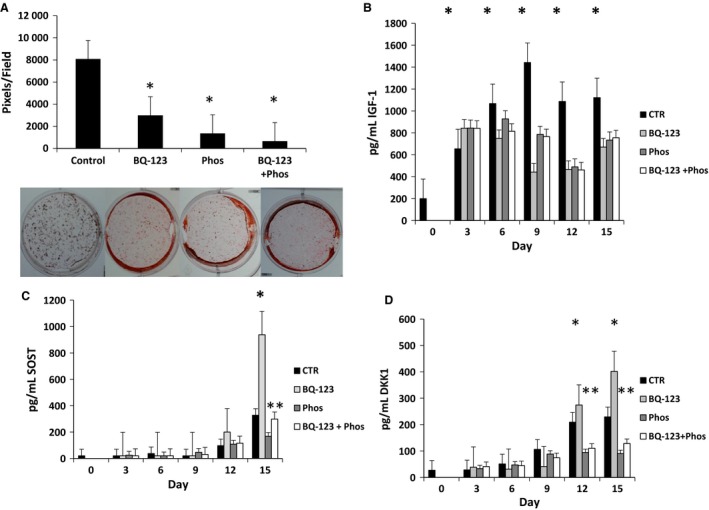
Mineralization and secretion of IGF1, DKK1, and SOST in TMOb cells in the presence of EDNRA and ECE‐1 inhibition. (A) Mineralization of TMOb cells in the presence of BQ‐123 (EDNRA inhibitor) and/or phosphoramidon (ECE‐1 inhibitor). Each image is a representative well cut from a picture of a six‐well plate that makes up a treatment. The presence of either BQ‐123 and/or phosphoramidon decreased mineralization of TMOb cells (*P* < 0.001) as measured by ANOVA. (B) Analysis by two‐way repeated‐measures ANOVA of IGF1 protein secretion in TMOb cells in the presence of BQ‐123 (EDNRA inhibitor), phosphoramidon (ECE‐1 inhibitor), and both phosphoramidon and BQ‐123. There were significant decreases in the treatment group versus control (*P* < 0.001). There were significant decreases in IGF1 secretion between the treatment group and control on days 3, 6, 9, 12, and 15 (*P* < 0.05). Analysis by ELISA. (C) SOST protein secretion in TMOb cells in the presence of BQ‐123 (EDNRA inhibitor), phosphoramidon (ECE‐1 inhibitor), and both phosphoramidon and BQ‐123. There were significant differences by two‐way repeated‐measures ANOVA between the control group and treatment groups in treatment over time (*P* < 0.001). We saw the predicted increases in the ENDRA inhibition group on day 15 (*P* < 0.05), but there was an unexpected decrease in secretion in the ECE‐1‐inhibited groups on day 15 (*P* < 0.05). Analysis by ELISA. (D) Analysis of DKK1 protein levels by two‐way repeated‐measures ANOVA in TMOb cells in the presence of BQ‐123 (ENDRA inhibitor), phosphoramidon (ECE‐1 inhibitor), and both phosphoramidon and BQ‐123. There were significant differences between the control group and treatment groups in treatment over time (*P* < 0.001). We saw the predicted increases in the EDNRA inhibition group on days 12 and 15 (*P* < 0.05), but there was an unexpected decrease in secretion in the ECE‐1‐inhibited groups also on days 12 and 15 (*P* < 0.05). Each time point within a treatment is the average of six samples for all panels, and secreted protein levels were analyzed by two‐way repeated‐measures ANOVA. Analysis by ELISA. IGF1, insulin‐like‐growth factor‐1; DKK1, dickkopf‐homolog‐1; SOST, sclerostin; TMOb, immortalized mouse pre‐osteoblasts; EDNRA, endothelin receptor A; ECE‐1, endothelin‐converting enzyme‐1; ANOVA, analysis of variance. * denotes statistically significant increase. ** denotes statistically significant decrease.

To determine whether ET‐1 signaling mediates mineralization in the absence of exogenous ET‐1, we cultured TMOb cells in the presence of the EDNRA antagonist BQ‐123 and/or the ECE‐1 inhibitor phosphoramidon. Inhibition of ET‐1 signaling, using EDNRA and ECE‐1 antagonists, reduced mineralization by 64% (BQ‐123), 83% (phosphoramidon), and 92% (BQ‐123+ phosphoramidon) as shown in Figure 3A (*P* < 0.001 for each). Each picture is a representative well cut from a picture of a six‐well plate that makes up a time point. Exposure to BQ‐123 and phosphoramidon decreased IGF1 secretion over time (*P* < 0.001). There were significant decreases in IGF1 secretion between the treatment group and control on days 3, 6, 9, 12, and 15 (*P* < 0.05) (Fig. 3B). Incubation of mineralizing TMOb cells with BQ‐123 increased SOST and DKK1 secretion compared with control (*P* < 0.05). In contrast, DKK1 secretion decreased and SOST secretion decreased or remained the same when TMOb cells were incubated with phosphoramidon (Fig. 3C and D). When DKK1 and SOST levels decrease or remain the same, we would expect similar or increased mineralization.

### Knockdown of *ECE‐1* using siRNA

**Figure 4:**
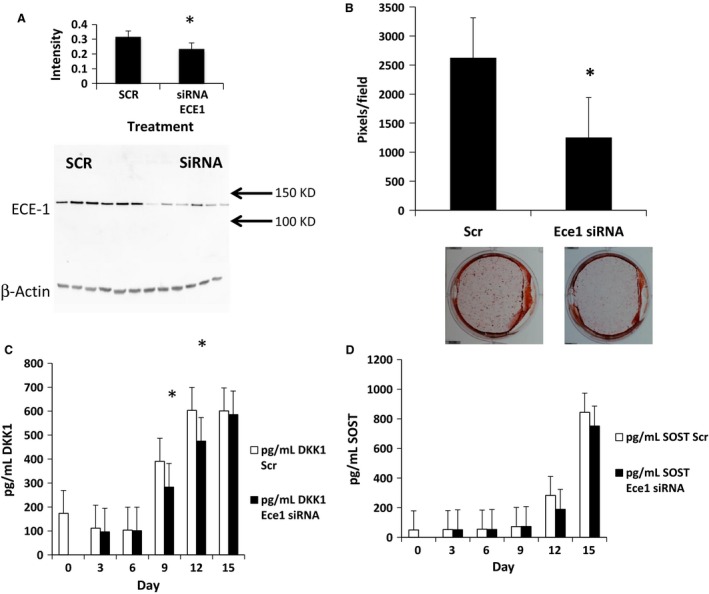
Mineralization and secretion of IGF1, DKK1, and SOST in TMOb cells following transfection with *ECE‐1* siRNA. SCR denotes scramble control. SiRNA denotes *Ece1* siRNA. (A) Nine days after the second transfection of ECE‐1 siRNA on day six, ECE‐1 production was still reduced by 30% on day 15 in the siRNA‐transfected cells (*P* < 0.05). Each time point is the average of six samples, and each group was analyzed by *t*‐test. The figure shows representative lanes from each set of six samples, using the same lane for the ECE‐1 and *β*‐actin. (B) Mineralization of TMOb cells after transfection with *ECE‐1* siRNA. Mineralization in the siRNA group was significantly less than the control group (*P* < 0.001) as analyzed by ANOVA. Each image is a representative well cut from a picture of a six‐well plate that makes up a treatment. (C) Analysis of secreted DKK1 protein in TMOb cells treated with *ECE‐1* siRNA. There were significant differences between the control group and the *ECE‐1* siRNA group in treatment over time (*P* < 0.001) as analyzed by two‐way repeated‐measures ANOVA. Analysis by ELISA. (D) Analysis of SOST protein levels in TMOb cells treated with *ECE‐1* siRNA. While there was a trend toward less SOST secretion (*P* = 0.087) in the treatment group, there were no significant differences between groups. Each time point within a treatment is the average of six samples for all panels and secreted protein levels were analyzed by two‐way repeated‐measures ANOVA. Analysis by ELISA. IGF1, insulin‐like‐growth factor‐1; DKK1, dickkopf‐homolog‐1; SOST, sclerostin; TMOb, immortalized mouse pre‐osteoblasts; ECE‐1, endothelin‐converting enzyme‐1; ANOVA, analysis of variance. * denotes statistically significant difference.

To determine whether the divergent secretory responses of IGF1 and WNT antagonists were ECE‐1 dependent, or a result of nonspecific metalloprotease inhibition by phosphoramidon, we transfected TMOb cells with *ECE‐1* siRNA to knock down ECE‐1 activity. Transfected cells showed a 59% decrease in *ECE‐1* transcript (*P* < 0.05) 3 days after the transfection on day 0. We also analyzed ECE‐1 protein levels on day 15 and found that there was still a 30% decrease in protein production (*P* < 0.005) on day 15 (Fig. 4A), 9 days after the second transfection. Mineralization was lower in *ECE‐1* siRNA‐treated cells (*P* < 0.001) (Fig. 4B). Each picture is a representative well cut from a picture of a six‐well plate that makes up a time point. In accord with pharmacological inhibition of ECE‐, *Ece‐1* siRNA‐treated cells, DKKI secretion was significantly lower over time (*P* = 0.005) with decreased secretion on days 9 and 12 (*P* < 0.05) (Fig. 4C). In contrast, while there was a trend toward decreased secretion, SOST secretion did not change significantly over time (Fig. 4D). Similar to our pharmacological results, TMOb cells with decreased or similar DKK1 and SOST levels should show similar or increased levels of mineralization, not the decreases seen in both sets of experiments. Our results suggest a potential role for ECE‐1 in osteoblast differentiation and mineralization beyond activation of ET‐1./

### Effect of overexpression of MiR 126‐3p and its inhibitor in TMOb cells

**Figure 5:**
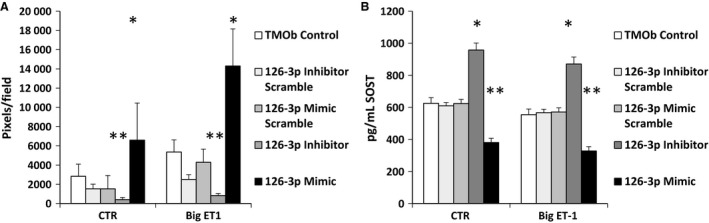
(A) Mineralization of TMOb cells harboring miR 126‐3p lentivirus constructs ± big ET‐1. The cell line expressing the miR 126‐3p inhibitor had significantly less mineralization in both treatments (*P* < 0.001), while the cell line expressing miR 126‐3p significantly increased mineralization (*P* < 0.01) by ANOVA. Each image is a representative well cut from a picture of a six‐well plate that makes up a treatment. (B) The 126‐3p inhibitor significantly increased SOST secretion in both treatment groups (*P* < 0.001), while the miR 126‐3p caused significant decreases in SOST secretion in both treatments (*P* < 0.001) as analyzed by ANOVA. Each time point within a treatment is the average of six samples. Analysis by ELISA. TMOb, immortalized mouse pre‐osteoblasts; ET‐1, endothelin‐1; ANOVA, analysis of variance; SOST, sclerostin. * denotes statistically significant increase. ** denotes statistically significant decrease.

Previously, we demonstrated that exposure of TMOb cells to big ET‐1 resulted in profound changes in the levels of multiple miRs, including miR 126‐3p which we identified as a potential regulator of SOST secretion (Johnson et al. 2014). We saw a 121X increase in miR 126‐3p levels in TMOb cells exposed to big ET‐1 during mineralization and a significant decrease in SOST secretion (Orzechowski et al. 1997). To test the hypothesis that miR 126‐3p regulates SOST production, we constructed stable TMOb cell lines expressing miR 126‐3p, its inhibitor, and two scrambles. We grew and mineralized these cell lines ± big ET‐1. Figure 5 shows that the cell lines transfected with miR 126‐3p displayed increased mineralization (*P* < 0.001) in both the presence and absence of big ET‐1 and decreased secretion of SOST (*P* = 0.007). The cell lines transfected with the 126‐3p inhibitor showed decreased mineralization (*P* < 0.001) and increased secretion of SOST (*P* < 0.001) both in the presence and absence of big ET‐1. These data show that one of the mechanisms by which big ET‐1 influences mineralization in TMOb cells is by regulation of miR 126‐3p levels that posttranscriptionally regulate production of SOST.

## Discussion

ET‐1 is an important molecule in physiology and development. Global ablation of any ET‐1‐related gene in mice is lethal in utero or perinatally (Kurihara et al. 1994; Clouthier et al. 1998; Yanagisawa et al. 1998; Kedzierski and Yanagisawa 2001). The developmental lethality of ET‐1 signaling axis‐deficient animals has hampered studies on the physiologic roles of ET‐1 signaling in bone. The only in vivo studies of ET‐1 signaling in bone utilized osteoblast‐specific *Ednra*‐ablated mice, which showed decreased trabecular volume (Clines et al. 2011). Research on osteoblastic metastases demonstrated a causal role for ET‐1 in woven bone formation and that blockade of ET‐1 signaling prevented disorganized bone growth in vivo (Guise et al. 2003, 2005, 2006; Mohammad and Guise 2003; Yin et al. 2003). In vitro, ET‐1 increased mineralization in osteoblast culture (Orzechowski et al. 1997; von Schroeder et al. 2003; Clines et al. 2007, 2011). However, the mechanisms by which ET‐1 increases mineralization and affects bone growth have not been defined. Two studies demonstrated that incubation of calvarial osteoblasts with ET‐1 increased translocation of *β*‐catenin to the nucleus, suggesting an increase in WNT signaling and that addition of the WNT inhibitor DKK1 blocked the ET‐1‐mediated increase in mineralization (Clines et al. 2007, 2011).

In our study, we demonstrated that treatment of TMOb osteoblasts with big ET‐1 decreased secretion of the WNT inhibitors SOST and DKK1, and increased secretion of IGF1. Big ET‐1 treatment did not alter osteoblast proliferation. The use of EDNRA, ECE‐1, or WNT inhibitors in the presence of big ET‐1 prevented the big ET‐1‐mediated increase in mineralization. Inhibition of EDNRA in the absence of exogenous big ET‐1 inhibited mineralization and disrupted normal secretion of SOST, DKK1, and IGF1. Inhibition of ECE‐1 pharmacologically or by siRNA also disrupted normal mineralization. IGF1 secretion decreased as expected suggesting an ECE‐1‐specific effect and not a nonspecific inhibition of multiple proteases by phosphoramidon. However, we saw an unexpected decrease in DKK1 and SOST secretion following ECE‐1 blockade, pharmacologically, and a significant decrease in DKK1 secretion and a numerical decrease in SOST secretion when *Ece‐1* is inhibited by siRNA. We believe that the lack of significance in the decrease in SOST secretion is due to the relatively poor efficiency of *Ece‐1* silencing. It is known that changes in SOST, DKK1, and IGF1 levels affect mineralization without concomitant changes in the levels of the other molecules (Liu et al. 1993; Li et al. 2005, 2008; He et al. 2006; Clines et al. 2007; Komatsu et al. 2010; Tu et al. 2012). It appears that mineralization is a more sensitive marker than changes in protein levels. While it could be argued that we should have done more silencing transfections, treatment of control cells with transfection reagents and no siRNA decreased mineralization by 50% relative to normal mineralization (Figs. 1A, 2A, 4A). It is possible that disruption of the plasma membrane by the transfection reagents disrupts the cell‐to‐cell communication needed for mineralization (Cheng et al. 2000; Furlan et al. 2001; Stains and Civitelli 2005).

Normally decreases in SOST and DKK1 secretion would increase mineralization. However, DKK1 and SOST levels are also markers of osteoblast maturity and the ability of the cells to mineralize tissue (Chen et al. 2015). Therefore, a possible explanation for these findings is impaired maturation of the TMOb cells following ECE‐1 inhibition. This raises the possibility of another, as yet unidentified, substrate of ECE‐1 involved in osteoblast maturation and WNT signaling regulation. Such a maturational block could account for the simultaneous decrease in mineralization, SOST, and DKK1 secretion.

Our data demonstrate that ECE‐1, EDNRA, and WNT signaling are required for the ET‐1‐mediated increase in mineralization. We demonstrated that secretion of IGF1, DKK1, and SOST are uncoupled from transcription in the presence of exogenous big ET‐1, suggesting a posttranscriptional regulatory mechanism. Previously, we showed that big ET1 increased levels of mir 126‐3p by 121x during mineralization (Kedzierski and Yanagisawa 2001). Further, we demonstrated that miR 126‐3p levels inversely correlated with SOST secretion but not *Sost* transcription and that increases in miR‐126‐3p levels increased mineralization. Conversely, inhibition of miR‐126‐3p decreased mineralization and increased SOST secretion. MiR 126‐3p was first identified as an important factor in angiogenesis (Fish et al. 2008; Kuhnert et al. 2008; Wang et al. 2008; Cao et al. 2015). MiR 126‐3p represses two negative regulators of VEGF signaling, sprouty‐related protein‐1, and phosphatidylinositol‐3‐kinase regulatory subunit 2, which leads to increased VEGF signaling and increased angiogenesis (Kurihara et al. 1994; Little et al. 2002a; Li et al. 2005, 2008). Similarly, SOST and DKK1 are negative regulators of WNT signaling. Our data show that, in bone, miR 126‐3p negatively regulates SOST. Increases in miR 126‐3p levels decrease levels of SOST secretion, which leads to increased WNT signaling and mineralization. ET‐1 signaling may also regulate IGF1 and DKK1 in a miR‐dependent manner, as our data show uncoupling of IGF1 and DKK1 transcription and secretion. The data suggest the existence of miR‐dependent, posttranscriptional regulation of big ET‐1 signaling in TMOb mineralization.

Previous genetic and biomechanical data (Orzechowski et al. 1997; Saless et al. 2009, 2010, 2011a,b; Kristianto et al. 2016) combined with the data reported here suggest a model in which modulation of ET‐1's activity by allelic variation of *Ece‐1* leads to changes in skeletal structure and biomechanical performance. Our study suggests that ECE‐1‐dependent ET‐1 signaling is involved in normal skeletal metabolism, and the ET‐1 axis modulates WNT signaling during bone formation. Previous studies have demonstrated the importance of WNT signaling in bone mass regulation and bones' response to mechanical loading (Robling et al. 2006; Sawakami et al. 2006; Tu et al. 2012). In particular, mice and humans carrying mutations in *Lrp5* that prevent SOST binding have increased skeletal mass (Little et al. 2002a,b; Sawakami et al. 2006; Niziolek et al. 2011). WNT signaling is required for skeletal response to mechanical load, and mechanical loading decreases SOST secretion (Robling et al. 2006, 2008; Sawakami et al. 2006; Niziolek et al. 2011). ET‐1 signaling is known to mediate mechanical stimuli in the development of the chick Purkinje system (Gourdie et al. 1998; Takebayashi‐Suzuki et al. 2000; Hall et al. 2003), and we have demonstrated that addition of exogenous big ET‐1 to the culture medium of ex vivo bone cores and mechanical loading evokes similar physical and biochemical responses (Meyer et al. 2016), so there are precedents for proposing that mechanotransduction in bone is also mediated by ET‐1. These data suggest a potential mechanism by which cross talk between ET‐1 and WNT signaling mediates the skeletal response to mechanical load.

Our study has several strengths. We used both ECE‐1 pharmacological inhibition and ECE‐1 siRNA knockdown to study ECE‐1‐mediated ET‐1 signaling in mineralization. We were able to demonstrate that knockdown of *ECE‐1* had similar results to pharmacological blockade of the protein. To further confirm the role of ET‐1 signaling in mineralization, we blocked EDNRA‐mediated signaling using an EDNRA‐specific antagonist in the presence of exogenous big ET‐1. We also demonstrated a mechanism by which ET‐1 regulation of miR 126‐3p regulates SOST and WNT signaling in TMOb cells.

The in vitro nature of the study is the main limitation of our study. As our experiments were performed in vitro, we were unable to address the effect of ET‐1 signaling in vivo. Several aspects of our findings require further study. We have shown that the secretion and transcription of IGF1 and DKK1 are uncoupled, suggesting that ET‐1 signaling regulates their secretion in a posttranscriptional manner. However, we have not yet determined the mechanism by which this occurs. It is possible that miRs other than miR 126‐3p are involved, but this remains to be demonstrated. It is known that SOST secretion is suppressed by mechanical loading (Robling et al. 2006, 2008; Sawakami et al. 2006; Tu et al. 2012), but while our data are suggestive, it remains unknown whether and to what extent ET‐1 signaling contributes to this phenomenon.

In summary, our data show that ET‐1 signaling regulates secretion of IGF1 and the WNT signaling inhibitors DKK1 and SOST. We demonstrated that ET‐1 influences SOST secretion through regulation of miR 126‐3p (Orzechowski et al. 1997). While it is clear that ET‐1 in excess can drive bone pathology (Guise et al. 2003, 2005, 2006; Mohammad and Guise 2003; Yin et al. 2003), our data demonstrate that ET‐1 is a critical autocrine regulatory molecule in normal osteoblast physiology. We demonstrated that ET‐1 autocrine signaling is essential for normal osteoblast differentiation and mineralization and that osteoblast‐derived ET‐1 is dependent on ECE‐1. Our current study demonstrates a mechanism explaining how allelic variation in the *ECE‐1* gene might generate bone size and biomechanical performance phenotypes in mice by regulating the secretion of WNT signaling inhibitors and IGF1.

## Conflict of Interest

None declared.
